# Developing an Implementation Framework for Web-Based Presence Technology Integration in Long-Term Care Homes

**DOI:** 10.1093/geroni/igaf122.446

**Published:** 2025-12-31

**Authors:** Anna Garnett, Halyna Yurkiv, Denise Connelly, Richard Booth, Lorie Donelle

**Affiliations:** Western University, London, Ontario, Canada; Western University, London, Ontario, Canada; Western University, London, Ontario, Canada; Western University, London, Ontario, Canada; University of South Carolina, Columbia, South Carolina, United States

## Abstract

Web-based presence technologies (WPT) have been recognized for their ability to mitigate social isolation and foster a sense of aging in place among older adults within long-term care homes. Despite ongoing interest in WPT use by a range of stakeholders, a guiding framework for its sustained implementation in long-term care homes is lacking. This multi-method study sought to develop an evidence-informed framework for integration of WPT within long-term care homes. Using a qualitative descriptive approach, semi-structured interviews were conducted with 12 long-term care home leadership personnel, 22 family members, 10 staff, and seven older adults. Additionally, documents such as long term-care policies and guidelines were examined. Multi-stage data analysis included: 1) directed content analysis guided by Technology Acceptance Model to examine older adults, family members, and staff experiences; 2) conventional content analysis of documents and leaders’ accounts. Findings were triangulated to develop an implementation framework highlighting factors to be considered to achieve successful WPT integration within long-term care homes. Facilitating factors included: training to enhance digital literacy of WPT end users, collaboration between long-term care homes and external companies for resource acquisition (devices, funding, human resources), and diverse applications of WPT use (i.e., social connectedness, telehealth). Potential barriers included: funding constraints and competing priorities within long-term care, lack of standardized organizational guidelines on WPT use, and usability challenges among end-users. Findings highlight the need for sustained funding opportunities, ongoing digital literacy programs for end-users, and standardized guidelines to ensure equitable and sustained integration of WPT across long-term care homes.

